# Neuroimaging in ADHD: How far are scanners from clinical psychiatry?

**DOI:** 10.1192/j.eurpsy.2021.225

**Published:** 2021-08-13

**Authors:** V. Pereira-Sanchez

**Affiliations:** 1 Child And Adolescent Psychiatry, NYU Grossman School of Medicine, New York, Spain; 2 Psychiatry And Medical Psychology, Clinica Universidad de Navarra, Pamplona, Spain

## Abstract

**Abstract Body:**

Decades of neuroimaging research in attention/deficit-hyperactivity disorder (ADHD) have yielded a few apparently firm findings and many open questions. The long-term objective of these efforts is to uncover the underlying brain pathophysiology of the disorder, to reveal reliable biomarkers of prognosis and treatment response, striving for personalized medicine. Unfortunately, neuroimaging research in ADHD and other psychiatric disorders is still unable to inform clinical practice. This presentation will provide an up-to-date overview of neuroimaging in ADHD, highlighting the most promising results and current challenges of structural and functional research with magnetic resonance imaging (MRI). Evidence from large, multicentric studies and from highly-sophisticated resting-state functional MRI techniques will be presented; methodological and reproducibility limitations in current literature will be introduced, and the way forward to bring this area of research closer to clinical practice with patients with ADHD will be discused. Dr. Pereira-Sanchez is conducting original research using resting-state functional MRI to study potential correlates of treatment response to stimulants in children and adolescents with ADHD; he has also recently published two literature reviews of MRI studies in ADHD.

**Disclosure:**

No significant relationships.

